# Biophysical Stress Responses of the Yeast *Lachancea thermotolerans* During Dehydration Using Synchrotron-FTIR Microspectroscopy

**DOI:** 10.3389/fmicb.2020.00899

**Published:** 2020-05-12

**Authors:** Antonio Anchieta Câmara, Thanh Dat Nguyen, Rémi Saurel, Christophe Sandt, Caroline Peltier, Laurence Dujourdy, Florence Husson

**Affiliations:** ^1^Univ. Bourgogne Franche-Comt, AgroSup Dijon, PAM UMR A 02.102, Dijon, France; ^2^SMIS beamline, Synchrotron SOLEIL, Gif-sur-Yvette, France; ^3^AgroSup Dijon, Service d’Appui à la Recherche, Dijon, France

**Keywords:** dehydration, yeast, *Lachancea thermotolerans*, S-FTIR, proteins, lipids

## Abstract

During industrial yeast production, cells are often subjected to deleterious hydric variations during dehydration, which reduces their viability and cellular activity. This study is focused on the yeast *Lachancea thermotolerans*, particularly sensitive to dehydration. The aim was to understand the modifications of single-cells biophysical profiles during different dehydration conditions. Infrared spectra of individual cells were acquired before and after dehydration kinetics using synchrotron radiation-based Fourier-transform infrared (S-FTIR) microspectroscopy. The cells were previously stained with fluorescent probes in order to measure only viable and active cells prior to dehydration. In parallel, cell viability was determined using flow cytometry under identical conditions. The S-FTIR analysis indicated that cells with the lowest viability showed signs of membrane rigidification and modifications in the amide I (α-helix and β-sheet) and amide II, which are indicators of secondary protein structure conformation and degradation or disorder. Shift of symmetric C–H stretching vibration of the CH_2_ group upon a higher wavenumber correlated with better cell viability, suggesting a role of plasma membrane fluidity. This was the first time that the biophysical responses of *L. thermotolerans* single-cells to dehydration were explored with S-FTIR. These findings are important for clarifying the mechanisms of microbial resistance to stress in order to improve the viability of sensitive yeasts during dehydration.

## Introduction

Dehydration processes are a crucial part of yeast industrial usage. These processes involve different steps that may interfere with the yeasts’ cellular activity. During biomass production, high concentration of solutes in the medium at the beginning of cell propagation causes osmotic stress (Pérez-Torrado et al., 2015). During the propagation of biomass, the consumption of nutrients induces nutritional stresses that affect several cellular functions and may lead to cell death (Gómez-Pastor et al., 2011). However, one of the most important stresses occurs during dehydration. Removal of water by dry-air and elevated temperatures generate modifications in the water potential of the medium and in the cells themselves, leading to a decrease in their volume and an increase of their contact surface with air (Beker and Rapoport, 1987; Gervais and Beney, 2001; Lemetais et al., 2012; Rapoport et al., 2016). All these modifications can induce the accumulation of reactive oxygen species (ROS), consequently, oxidative stress and cause the cells death (Eleutherio et al., 2018).

The use of yeast *Lachancea thermotolerans* is currently of great interest for applications in various areas of the food and beverage industries (Vilela, 2018; Porter et al., 2019), such as the production of high lactic acid content beverages (Benito et al., 2017; Benito, 2018), the bio-sorption of ochratoxin A in wines (Morata et al., 2018) the development of new sensorial profiles in sour beers (Domizio et al., 2016) and the high resistance to freezing (Hino et al., 1990). However, this yeast is highly sensitive to dehydration (Rodríguez-Porrata et al., 2011; Câmara et al., 2019a). In this way, it is important to explore what biophysical modifications occur during dehydration that make this yeast particularly sensitive. Indeed, obtaining accurate biophysical and physiological information using dehydrated yeast cells is a challenge, especially when the interest is to understand the in-depth cellular biophysical modifications without a rehydration step. Most protocols of enzymatic activity determinations and measurements of proteins or lipids contents involve a liquid suspension of the cells or their cell extracts. During rehydration, multiple damage to intracellular compartments and/or sensitive molecules may occur, which may result in loss of lipids and proteins during extraction, also due to lipolytic and proteolytic activities of the cells (Ami et al., 2014).

Fourier-Transform Infrared (FTIR) spectroscopy is a non-invasive method used to analyze several materials, including biological samples. This method allows obtaining accurate data related to the macromolecular composition from their intrinsic vibrational energy states without staining or reagent, including lipids and proteins (Tamm and Tatulian, 1997). Specific absorption bands related to the lipids – CH_2_ *asym.* (2915 cm^–1^), CH_2_ *sym.* (2840 cm^–1^), CH_3_ *asym.* (2960 cm^–1^), and CH_3_ *sym.* (2875 cm^–1^), lipid ester C = O (1740 cm^–1^) – and to the proteins – amide I (1650–1660 cm^–1^), amide II (1545 cm^–1^) are frequently studied. The amide I band is generally decomposed in several peaks characteristic of the different protein conformations: α-helix (1656 cm^–1^), random coils (1635–1640 cm^–1^), and β-sheet (1627–1635 cm^–1^) (Barth, 2007; Coe et al., 2015). However, the biological samples used in conventional FTIR spectroscopy consist of a large population of cells, which gives global information on cell biophysical changes, but not at the single-cell level. FTIR microspectroscopy – the combination of an infrared microscope and a FTIR spectrometer – allows measuring the chemical composition of yeast microcolonies made of a few thousand cells (Wenning et al., 2002; Essendoubi et al., 2005; Toubas et al., 2006). Synchrotron-FTIR (S-FTIR) microspectroscopy allows both individual cell selection and obtaining the precise spectrum of the single yeast cell with a high signal to noise ratio thanks to the high brilliance of the synchrotron light source (Saulou et al., 2010; Jamme et al., 2013; Tobin et al., 2016). The combination of S-FTIR and fluorescence microscopy techniques on the same instrument makes it possible to selectively measure the infrared spectra of specific subpopulations of yeast cells marked by fluorescent stains or antibodies without prior sorting. Recently, using S-FTIR technique, the cell biophysical modifications were analyzed during dehydration of the yeast *Saccharomyces cerevisiae* at single-cell level (Nguyen et al., 2017) and during freezing of lactic acid bacteria (Passot et al., 2015).

In this work, we compared the single-cell biophysical responses of one *L. thermotolerans* strain after two different dehydration processes. Dynamic dehydration kinetics coupled to the S-FTIR and fluorescence microscopy permitted to evaluate *in situ* the evolution of the cell’s biophysical behavior before and after dehydration. In parallel, cell viability was determined by flow cytometry in order to correlate the obtained spectral data with cell survival or death.

## Materials and Methods

### Yeast Strain, Media Composition and Culture Conditions

The yeast strain studied was *L. thermotolerans* CBS 6340. Cells were grown in GSM (For 1 L: 30 g, glucose; 30 g, yeast extract; 0.6 g, KH_2_PO_4_; and, 0.6 g, cysteine) (Câmara et al., 2019a). Three previously isolated colonies onto YPD agar (For 1 L: 10 g, yeast extract; 20 g, peptone; 20 g, glucose; and, 15 g, agar) were grown in a 250 mL conical flask containing 50 mL of GSM and incubated in a rotary shaker at 140 rpm, 30°C for 24 h. The cells at stationary phase were then harvested by centrifugation (2,200 × *g* for 5 min) at 4°C. Cells were washed twice with acetate buffer solution (pH = 5.12) and suspended in the same buffer (final cell density approximately 2 × 10^10^ cells mL^–1^) before drying kinetics.

### Cell Preparation and Staining

**FIGURE 1:**
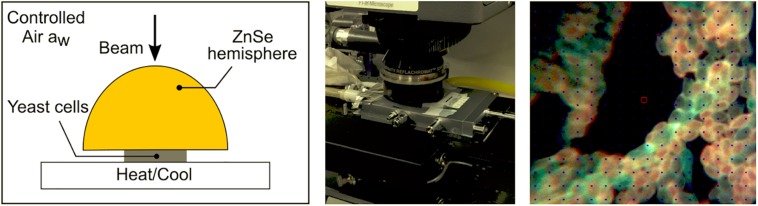
Schematic representation of the ZnSe hemisphere (left), images of the stage coupled to the FTIR microscope (center), the double-stained yeast *L. thermotolerans* cells (right). The cells were fixed on the surface of the ZnSe hemisphere and carefully placed on the base of the stage. The synchrotron beam was calibrated in the center of the hemisphere using the FTIR microscope. The air relative humidity and temperature were controlled during dehydration kinetics and acquisition of spectra. Only living (green) cells were selected before dehydration.

In order to measure only the living cells at the end of the dehydration process, cells were stained with two fluorescent dyes: fluorescein diacetate (FDA, green) (Sigma-Aldrich, St Louis, MO, United States) and propidium iodide (PI, red) (Sigma-Aldrich, St Louis, MO, United States). FDA is a metabolic cell enzymatic activity indicator and PI indicates the membrane integrity (for 10^8^ cells the final concentration was 10 μg FDA and 2 μg PI). An aliquot (2 μL) of double-stained cell suspension was deposited on the surface of the ZnSe hemisphere and held at controlled room temperature and relative humidity (RH) during 10 min (25°C and 23% RH) to attach cells on the hemisphere surface. Cells were then partially rehydrated in a hermetically box containing saturated water vapor during 60 min at 25°C before turning it upside down (Figure 1, left). The partial rehydrated cells attached to the ZnSe hemisphere were transferred to a Linkam FTIR-600 stage mounted on the motorized stage of a Continuum infrared microscope (Thermo-Fisher, Waltham, MA, United States), for spectral acquisition and fluorescence microscopy (Figure 1, center). In order to observe the effect of conservation or loss of viability, green-stained (live) cells were selected for spectral acquisition (Figure 1, right).

### Synchrotron FTIR Micro-Spectroscopy and Dehydration Kinetics

**FIGURE 2:**
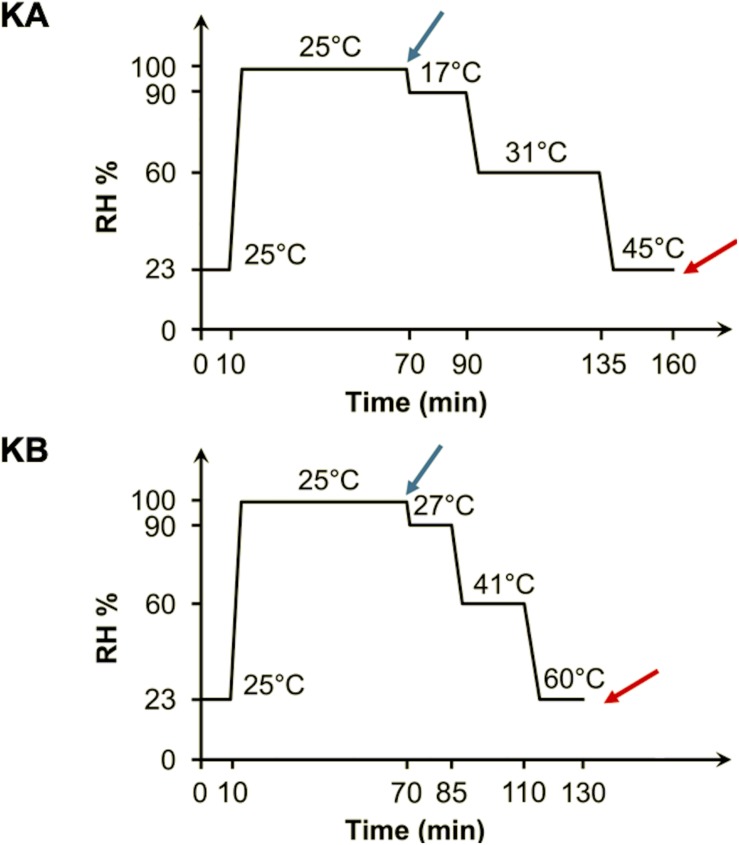
Dehydration kinetics parameters applied to yeast cells. Double-stained yeast cells were initially fixed (10 min at 23% RH and 25°C) and partially rehydrated (60 min at 99% RH and 25°C) before both KA and KB kinetics. For the KA kinetics, the cells were maintained for 20 min at 17°C and 90% RH, followed by 45 min at 31°C and 60% RH, and finally 25 min at 45°C and 23% RH. For the KB kinetics, the cells were maintained for 15 min at 27°C and 90% RH, followed by 25 min at 41°C and 60% RH, and finally 20 min at 45°C and 23% RH. The spectra were acquired before (blue arrow) and after (red arrow) the dehydration kinetics.

In order to observe biophysical profiles of yeast cells, S-FTIR measurements were carried out with same detailed protocol described by Nguyen et al. (2017) at a single-cell level. Control cells (C1 and C2) consisted of a population submitted to attachment and partial rehydration steps. At least 30 live cells were selected for spectral acquisition before initiating the dehydration process. The dehydration was carried out by flowing air at specific RH and temperature inside the FTIR-600 stage. Two different dehydration kinetics, named KA and KB, were performed as shown in Figure 2. These kinetics allowed to reproduce the dehydration processes applied in our previous work (Câmara et al., 2019b). A RH-temperature data logger was used to check the stability of the desired parameters during whole process.

The S-FTIR measurements were performed at the SMIS beamline of the SOLEIL synchrotron radiation facility. The SMIS beamline exploits the edge and constant field radiation from a bending magnet. The synchrotron delivered 430 mA and was operated in top-up mode. Infrared spectra were measured in reflection mode through a 10 mm diameter ZnSe ATR hemisphere with the Continuum microscope coupled to a Nicolet 8700 spectrometer. The spectrometer was equipped with a Michelson interferometer and KBr beamsplitter. The microscope was equipped with a Prior motorized stage, a 32X 0.65 NA Schwarzschild objective, and a narrow band MCT detector. The confocal aperture of the microscope was set at 12 × 12 μm^2^ which, projected on the sample through the ZnSe ATR hemisphere with a refractive index of 2.4, covered a 5 × 5 μm^2^ area at the hemisphere lower surface, approximately the size of one *L. thermotolerans* cell. Spectra were recorded between 4000 and 850 cm^–1^ at 6 cm^–1^ resolution with 256 scans and frequency of data record ∼2 cm^–1^. For fluorescence microscopy, the Continuum microscope was equipped with an Olympus fluorescence accessory fitted with an Ushio USH102D mercury lamp, and U-MWU, U-MWB, and U-MWG cube-corner filters.

### Assessment of Yeast Cell Viability Using Flow Cytometry

In order to evaluate cell viability, the dehydration kinetics (KA and KB) were reproduced inside of hermetically sealed drying chambers using different saturated salt solutions of potassium nitrate, sodium bromide or potassium acetate to control RH (Câmara et al., 2019a, b). These salts correspond to 90%, 60%, and 23% of RH, respectively (Greenspan, 1977; Labuza et al., 1985). Cells were previously prepared with the same staining-attachment-partial rehydration protocol described above. Initially, an aliquot (2 μL) of cell suspension was placed on a sterile polypropylene support, which was transferred to the first drying chamber, kept under controlled RH-temperature. It was then transferred successively at end of each cycle until the end of the kinetics (Figure 2) and supports were transferred to 15 mL tube. Cells were rehydrated with 5 mL of acetate buffer (pH 5.12) at 38°C during 5 min, vigorously stirred during 30 sec (final cell density 10^7^ cells mL^–1^) and immediately analyzed. Cell viability measurements were assessed using a BD FACS Aria II flow cytometer analyzer (BD Biosciences, San Jose, CA, United States) equipped with two lasers (excitation lines at 488 nm and 633 nm). The FDA and PI fluorescence signals were detected with at least 10,000 events in each analysis and data was compensated based on the staining of each individual fluorochrome alone and corrected for autofluorescence with unstained cells. The cell viability data significant differences (*n* = 3) were analyzed by the Tukey HSD *post-hoc* test using the STATISTICA^®^ software (Statsoft, Tulsa, OK, United States).

### Spectral Data Analysis

**FIGURE 3:**
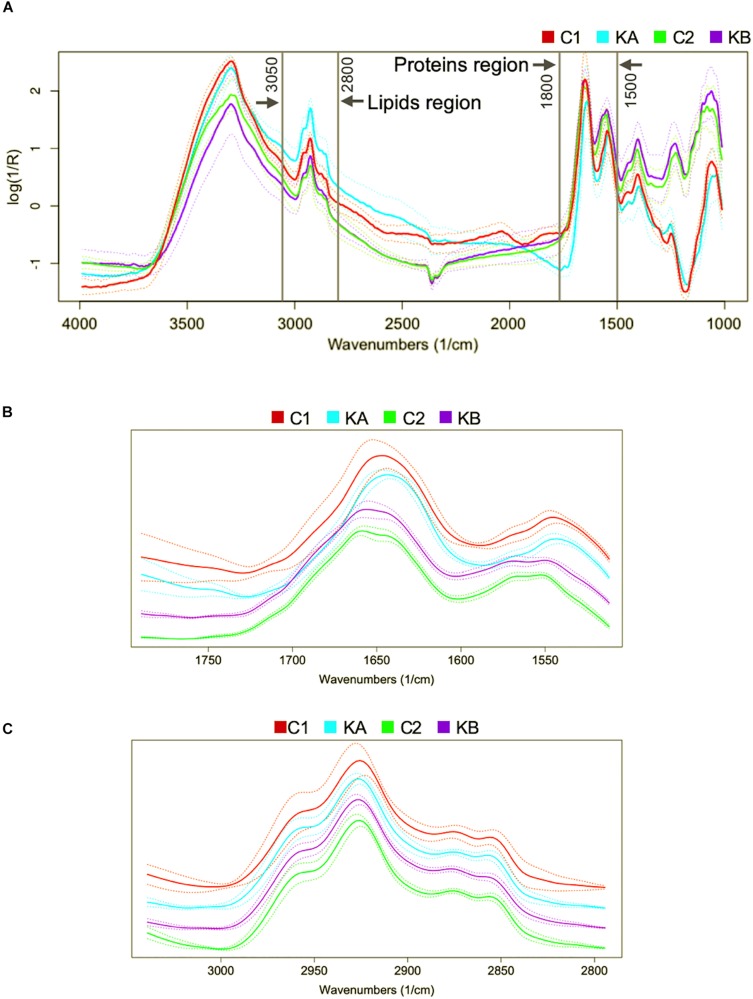
Representative FTIR raw spectra in the 4000–850 cm^–1^ region of the yeast *Lachancea thermotolerans* single-cells. The interest molecular group vibrations are indicated as the spectral regions of the proteins (1800–1500 cm^–1^) and lipids (3050–2800 cm^–1^). **(A)** Raw mean spectra for each performed condition. **(B)** Mean treated spectra for each performed condition in the protein region. **(C)** Mean treated spectra for each performed condition in the lipid region. For more clarity spectra were offset.

In order to analyze the effects of dehydration on the biophysical modifications of the yeast *L. thermotolerans* two regions were defined: (i) the region of the proteins absorption bands from 1800 to 1500 cm^–1^, which is characterized by conformation of amide I and amide II, β-sheet, random coils, and α-helix bands (Nguyen et al., 2017; Figure 3A); and (ii) the region of the lipid absorption bands from 3050 to 2800 cm^–1^, this region is characterized by vibrations of various fatty acid chains, phospholipids and some amino-side chains vibrations (Passot et al., 2015), as shown in Figure 3A.

After spectra acquisition, mathematical pre-treatments are necessary in order to eliminate experimental biases in spectral data. The classical pre-processing techniques are baseline correction, normalization, derivatives, and smoothing (Beebe et al., 1998; Wehrens, 2011). Combinations of these techniques were computed in this order: (1) baseline correction (for reducing systematic variation from background signal) (2) Savitzky-Golay (SG) filters (for reducing the amount of random variation such noise) and (3) normalization to unit vector (for removing systematic variation associated with the total amount of the sample). With SG pre-treatment, each point is replaced by a smoothed estimate obtained from a local polynomial regression. The choice of two parameters is required: *w* corresponding to the window size in polynomial smoothers, and *p* the polynomial order. In this study, the optimal window size and polynomial order were obtained with *w* = 13 and *p* = 3. All these analyses were computed using R 3.3.2 (R Core Team, 2018). The package prospectr (Stevens and Ramirez-Lopez, 2013) was used for SG and the package hyperSpec (Beleites and Sergo, 2018) was used for the other pre-treatments. Figures 3B,C show the pre-processed average spectral data (continuous curves) and their deviations (dotted curves) corresponding to the two defined regions: proteins and lipids, respectively.

Principal component analysis (PCA) was performed on the second derivatives of pre-processed spectral data and mean values of these respective second derivatives in the regions of interest were plotted. The PCA corresponds to a linear combination of the spectral data of uncorrelated variables. The first principal component (PC1) reflects the maximum variance of the data, and the second principal component (PC2) corresponds to the maximum possible variance remaining (orthogonal) (Cavagna et al., 2010). In addition, the most prominent peaks of the loading plots of each principal component represent variations in the most significant absorption bands. PCA was performed using the prcomp algorithm computed using R 3.3.2 (R Core Team, 2018).

## Results and Discussion

### Cell Viability and Biomass

**FIGURE 4:**
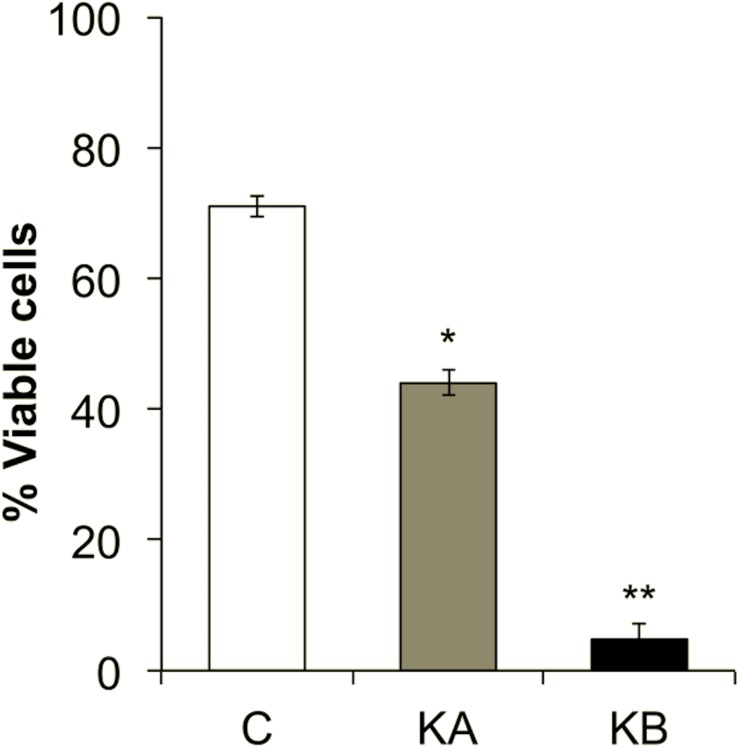
Cell viability of the *Lachancea thermotolerans* yeast strain CBS 6340. Yeast cells were harvested at the stationary phase and dehydrated according to the kinetics KA or KB. A microbial suspension submitted to the attachment-rehydration stages, but not to dehydration step, was used as control (C). Significant differences with the corresponding control condition were tested according to Tukey’s HSD *post-hoc* at: *p < 0.05 or **p < 0.01.

The viability of the yeast *L. thermotolerans* cells was affected by dehydration kinetics. Prior to dehydration, yeasts showed 71% viability (Figure 4). When cells were dehydrated with kinetics KA, they showed ∼44% of cell viability and <10% after kinetics KB (Figure 4). These results suggest a strong cell survival/death behavior depending on applied kinetics. It was previously shown that *S. cerevisiae* yeast cells grown in nutrient-rich media were more resistant to dehydration performed in a fluidized bed (Câmara, 2018) or *L. thermotolerans* cells under controlled air humidity (Câmara et al., 2018, 2019b) both in a dehydration temperature dependent manner. The loss of cell viability was in part attributed to the large amount of glutathione disulfide (oxidized glutathione) produced by the yeast cells during dehydration with kinetics KB, in comparison to kinetics KA. In the present study, the attachment of the cells on the ZnSe hemisphere before S-FTIR analysis included a partial attachment-rehydration step during 70 min and may have induced of the thermo-oxidative resistance decrease of the *L. thermotolerans* strain. Indeed, it was observed that the cells of yeast *L. thermotolerans* grown under identical conditions, but not submitted to this attachment-partial hydration step, preserved cell viability by ∼95% (Câmara et al., 2019a). Moreover, increasing contact time of the cells with the drying air increases the yeast oxidative stress, which partially explains the reduced cell viability in the control samples (71%) (Câmara et al., 2019b). It was shown with different wine yeast *S. cerevisiae* strains that a dehydration cycle of 24 h at 30°C and 8% RH caused from 25 to 65% of cell death according to the strain (Gamero-Sandemetrio et al., 2014). These observations were mostly attributed to modifications in the intracellular redox environment of cells (glutathione, catalase activity, and lipid peroxidation levels). In addition, increasing air temperature during dehydration with KB kinetics up to 60°C may have affected a number of physiological mechanisms essential for the metabolic activity of yeast *L. thermotolerans*. It was well demonstrated with the yeast *S. cerevisiae* that different temperature gradients, slope with rate of 0.5°C min^–1^ and shock with rate of 33°C min^–1^, up to 50°C reduced cell viability from 80% (slope) to 12% (shock), these results were correlated to modifications on yeast membrane permeability/fluidity that were also highly strain dependent (Guyot et al., 2015). Also, it was previously shown that *L. thermotolerans* cells grown in nutrient-rich conditions accumulate much more glutathione (mostly in the reduced form), reflecting in better survival rates after dehydration at 45°C and 90 min in laboratory scale (Câmara et al., 2019a).

The drastic reduction of *L. thermotolerans* cell viability after dehydration with KB kinetics (<10%) (Figure 4) may be related to the impact of the temperature gradient applied in this condition. Indeed, modification in the temperature of the cellular environment has two consequences: water transfer (fast) and heat transfer (slower), these two disturbances create an extra and intracellular osmotic gradient that affect cell viability (Gervais and de Marañon, 1995). Sudden intracellular water transfer can induce cell death mainly due to reduced cell volume, increased osmotic gradient and temperature (Gervais and Marechal, 1994). For example, a 4-fold increase in *D*-value (thermal death time) was observed when *S. cerevisiae* yeast cells were dehydrated at 45°C in a fluidized bed dryer, compared to 60°C, i.e., yeast cells were less resistant to higher temperature (Bayrock and Ingledew, 1997). Although distinct dehydration conditions, these phenomena help to clarify the higher cell viability (44%) after dehydration kinetics KA in comparison to KB (<10%) (Figure 4).

**FIGURE 5:**
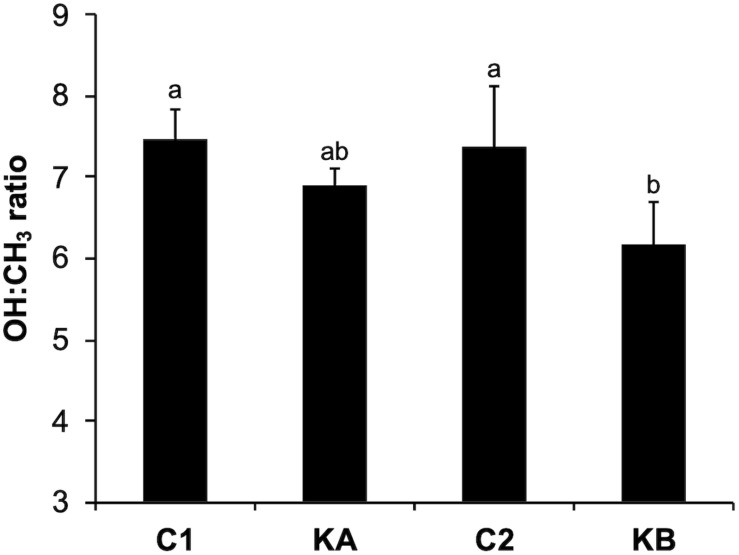
Water-biomass ratio of yeast *L. thermotolerans* before and after dehydration kinetics KA and KB. Data were calculated as the ratio between the OH (∼3400 cm^–1^) and CH_3_ (∼2960 cm^–1^) content. ^ab^Different letters indicate significant differences according to Tukey’s HSD *post-hoc* at *p* < 0.05.

The rates between the OH (3400 cm^–1^) and CH_3_ (2960 cm^–1^) bands were determined by integrating the peaks of the raw spectra of these regions of interest. The OH band indicating the intracellular water content was determined between 3400 and 3500 cm^–1^ and the CH_3_ band indicating the protein content between 2940 and 2950 cm^–1^. Thus, the OH:CH_3_ ratios provide information on the cellular water:biomass content and are represented in Figure 5. It can be noted that the values were not significantly different for the control conditions, C1 and C2 (7.47 and 7.39, respectively). After dehydration with KA kinetics the OH:CH_3_ content was 6.91, showing a slight reduction in the amount of water. The kinetics KB was more impacting on the reduction in the OH:CH_3_ ratio with a value of 6.16. This was expected due to the higher temperature of the kinetics KB, which leads to more pronounced water loss. These findings correlated with the cell viability indices found for both KA and KB kinetics.

Nevertheless, other mechanisms may govern yeasts survival/death as a function of temperature such as: water reactivity or mobility, thermostability of proteins, lipids and sugars present in the cell, the synthesis of heat stress proteins (HSP), redox reactions, among others (Dupont et al., 2014). Thus, these phenomena do not occur alone but in a complex network of biophysical stress response interactions. Further studies should focus on determining the *D*-value and *Z*-value for this sensitive *L. thermotolerans* strain and others.

### Evolution of Protein Profiles of Yeast *L. thermotolerans* During Dehydration

In order to evaluate modifications of *L. thermotolerans* cells biophysical profiles during dehydration, IR spectra from individual yeast cells were acquired before and after dehydration and PCA was performed on processed spectral data (base-line correction, smoothing with SG treatment, normalization and second derivative), as described above. PCA is a multivariate analysis method that can be used for discriminating patterns and clusters in FTIR data, as well as to identify specific spectral regions (absorption peaks) that contribute to the differences between the populations analyzed. Two spectral regions were defined: (i) protein region with wavelength between 1800 and 1500 cm^–1^, and (ii) lipid region with wavelength between 3050 and 2800 cm^–1^.

**FIGURE 6:**
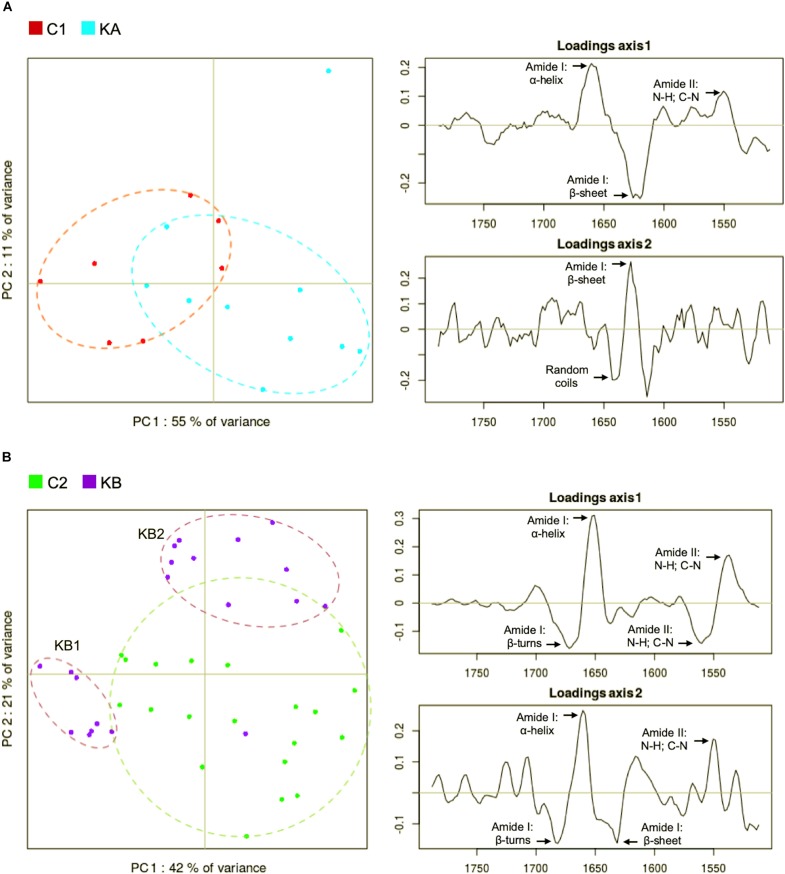
Principal component analysis (PCA) of second derivative infrared spectral data in the protein region (1800 to 1500 cm^–1^). Spectral data was acquired before and after dehydration with kinetics KA and KB. C1 and C2 correspond to partially hydrated cells before KA and KB kinetics. **(A)** Principal component 1 (PC1) vs. principal component 2 (PC2) score plots and the loading plots for axis 1 (PC1) and axis 2 (PC2) for KA dehydration kinetics; **(B)** Principal component 1 (PC1) vs. principal component 2 (PC2) score plots and the loading plots for axis 1 (PC1) and axis 2 (PC2) for KB dehydration kinetics.

Figure 6 present the PC1 vs. PC2 (the first principal component vs. the second) score plots and their respective loading plots (loadings axis 1 corresponds to PC1 and loadings axis 2 corresponds to PC2) for the second derivatives in the protein spectral region which may give information on the integrity/loss of integrity of the protein conformation. Figures 6A,B correspond to dehydration kinetics KA and KB, respectively. C1 and C2 groups are the control spectra measured on partially hydrated cells prior to KA and KB dehydration kinetics.

The PCA score plot show that the C1 and the KA clusters separated almost completely along PC1 axis (Figure 6A, left). The main spectral contributions in the loadings in the protein region of the spectra were the amide I and II, in the α-helix, random coils and β-sheet bands (Figure 6A, right). The loadings of the PC1 axis (Figure 6A, right) separating the C1 and KA clusters shows strong spectral weights at 1656 cm^–1^ (α-helix) and 1627 cm^–1^ (β-sheet) evidencing a shift of the amide I band toward lower (1656 ≥ 1627) wavenumbers upon dehydration of KA.

In Figure 6B, it can be seen that the KB spectra formed two clusters (purple dots), one located on the left along PC1 axis and the second located on the upper right region. The C2 cluster was fully separated from the second KB cluster on the PC2 axis but only partially separated from the first KB cluster on PC1 axis. Also, higher variability (plots dispersion) was detected for cells prior to dehydration (control cells C2, green plots), Figure 6B. For the two KB spectra clusters, we hypothesize that it could be an effect of the continuing evolution of the sample after the end of the dehydration kinetic. The first KB cluster regroups spectra from the eight first measured cells while the second KB cluster regroups the rest of the cells at the end of the measurement. At the end of dehydration kinetics, the spectral data acquisition was not simultaneous for all cells, indeed it took about 15 min for each point and cells remained in the dehydration conditions until the end of data capture. In this case, the first eight spectra corresponded to the first 120 min after the end of the kinetic. From this point in time, a clear cluster separation was observed (Figure 6B). PC1 and PC2 loadings both captured changes in the amide I band profile that explain the clustering of KB and C2 cell spectra. The first group KB1 (left on the PCA) separated by both PC1 and PC2 show a strong contribution of α-helix (1653 cm^–1^) and a shift of amide band II upon low wavenumbers. The second group KB2 (top-right on the PCA) separated by PC2 show an evidence of the amide I band reduction.

We observed different phenomena depending on the applied dehydration kinetics. Firstly, the controls showed distinct spectral differences between KA and KB, which suggests an experimental variability inherent to the studied strain. For the KA group, modifications of the amide I band to lower wavenumbers suggests the appearance of β-sheets and changes of protein conformation. For the KB group, two independent effects were observed. In the early stages, the KB1 group showed an amide II band shift toward low wavenumbers and modification of β-turns. For the KB2 group, continued exposure to dehydration induced an enlargement of amide I band and the appearance of β-turns and β-sheet bands but in a less evident manner than in KA. Higher temperature may have induced modifications in protein conformation for cells that remained in the stressful environment (final stage at 60°C and 23% RH) and this also partially explains the reduction of ∼60% on cell viability observed for cells dehydrated with kinetics KB, in comparison to cells dehydrated with kinetics KA (45°C and 23% RH) that have lost ∼27% (Figure 4). Indeed, several studies described the association of modifications in α-helix and β-sheet structures with the cell death (Zhou et al., 2001; Jamin et al., 2003; Zelig et al., 2009). The overall contribution of amide I spectral vibration can be explained by the protein degradation after dehydration (Burattini et al., 2008). The biophysical changes observed in the KB group give strong evidence of the harmful effects of dehydration that lead to cell death. However, in the KA group, the presence of about half of viable cells after dehydration leads us to believe that these biophysical modifications are signs of viability maintenance. These findings indicate that modification of the secondary proteins structure may be an indicator of the stress response of the *L. thermotolerans* strain.

**FIGURE 7:**
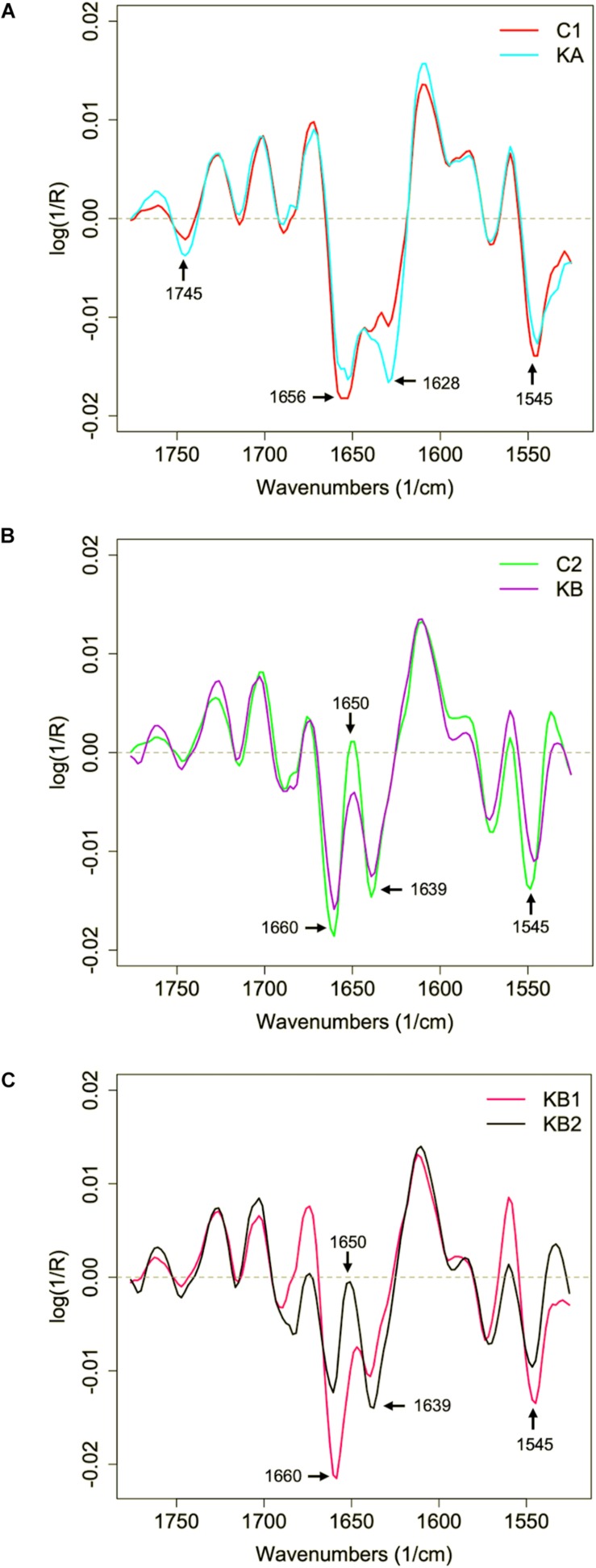
Second derivative of the mean infrared spectral data of the yeast *L. thermotolerans* protein region before and after dehydration. Cells were dehydrated according to kinetics KA and KB. **(A)** Protein spectral profile of yeast cells before (red curve, C1) and after dehydration with kinetics KA (cyan curve). **(B)** Protein spectral profile of yeast cells before (green curve, C2) and after dehydration with kinetics KB (purple curve). **(C)** Protein spectral profile of KB clusters KB1 (pink curve) and KB2 (black curve).

Regarding the mean second derivative plots of protein region spectral data for C1/KA and C2/KB conditions (Figures 7A,B), a clear vibration modification was observed in the amide I region: 1650–1656 cm^–1^ (α-helix), 1628–1639 cm^–1^ (β-sheet), and amide II region: 1545 cm^–1^ (N-H; C-N) before and after dehydration kinetics. Modifications were observed in the β-sheet band (1628 cm^–1^) when the cells were dehydrated with kinetics KA (Figure 7A). This observation suggests variations in the amide I band, which is affected by the nature of the secondary protein backbone structure (Barth, 2007). Changes were also observed at 1745 cm^–1^ bands (Figure 7A), where the C = O stretching of esters functional groups in lipids (mainly phospholipids) contributes to the vibrations in this region (Socrates, 2004) which was reported as an indicator of early cellular apoptosis (Jamin et al., 2003).

With respect to C2/KB condition (Figure 7B), besides the modifications in the β-sheet (1639 cm^–1^) absorption band, the effect of vibration in the 1650 cm^–1^ region was also observed after dehydration with kinetics KB (Figure 7B), this region corresponds to the contributions of the α-helix group. It was demonstrated with the yeast *S. cerevisiae* that cells dehydrated at 23% RH and 45°C showed modifications of the α-helix band of the proteins, which correlated with a higher cell mortality (approximately 1.5 log reduction) in comparison to cells dehydrated at same RH but at 25°C (Nguyen et al., 2017). In order to clarify the KB groups analyzes, the mean second derivative in the protein region were separated for KB1 and KB2 clusters (Figure 7C). For the first cluster KB1, it was observed that the amide I peak shifts to 1660 cm^–1^ while the β-sheet band (1639 cm^–1^) is reduced. Also, a strong contribution of 1545 cm^–1^ band was observed for the first cluster KB1. The KB2 cluster showed high contribution of β-sheet (1639 cm^–1^) and a reduction in the α-helix (1650 cm^–1^) band. These observations confirm the cluster observed in PCA analysis and demonstrate the impact of continuous KB kinetics on physiological modifications of *L. thermotolerans* yeast. However, it was not clear why the α-helix does not appear in the control samples in C1 condition.

The changes in the amide I zone suggest modification in the individual absorptions of α-helix and β-sheet, related to the transition from partially degraded proteins to an unfolded and disorganized state (Burattini et al., 2008). The amide II region has an important contribution of the curvature of the N-H bond and a small contribution of the C-N bond (Delgado et al., 2018). In this case, these modifications were most probably a consequence of protein degradation and transition of the protein to an unfolded state (Kavosi et al., 2018). These changes explain the significant loss of viability, from ∼71% to <10%, of L. thermotolerans CBS 6340 strain after dehydration with kinetics KB. Other cell models seem to have similar biophysical mechanisms during environmental stresses. For example, freezing resistant bacteria *Lactobacillus delbrueckii* ssp. *bulgaricus* showed higher β-sheet secondary structure absorption peak and with ∼20% loss of cultivable cells after freezing-thawing, while fresh cells showed higher intensity in α-helix secondary structure band (Passot et al., 2015).

### Evolution of Lipids Profiles of Yeast *L. thermotolerans* During Dehydration

**FIGURE 8:**
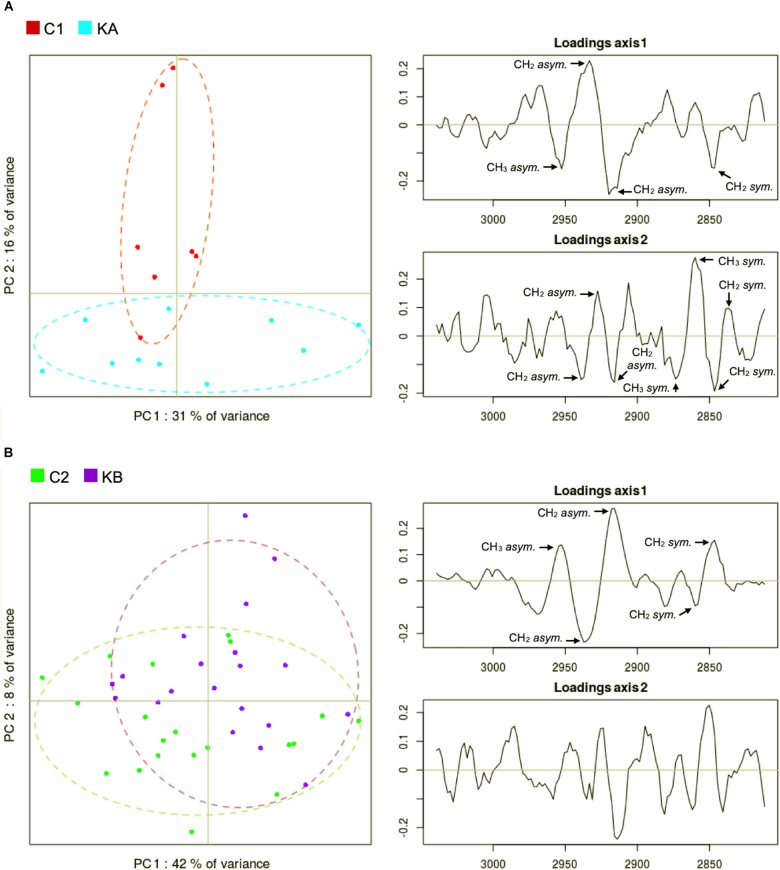
Principal component analysis (PCA) of second derivative infrared spectral data in the lipid region (3050 to 2800 cm^–1^). Spectral data was acquired before and after dehydration with kinetics KA and KB. C1 and C2 correspond to partially hydrated cells before KA and KB kinetics. **(A)** Principal component 1 (PC1) vs. principal component 2 (PC2) score plots and the loading plots for axis 1 (PC1) and axis 2 (PC2) for KA dehydration kinetics; **(B)** Principal component 1 (PC1) vs. principal component 2 (PC2) score plots and the loading plots for axis 1 (PC1) and axis 2 (PC2) for KB dehydration kinetics.

**FIGURE 9:**
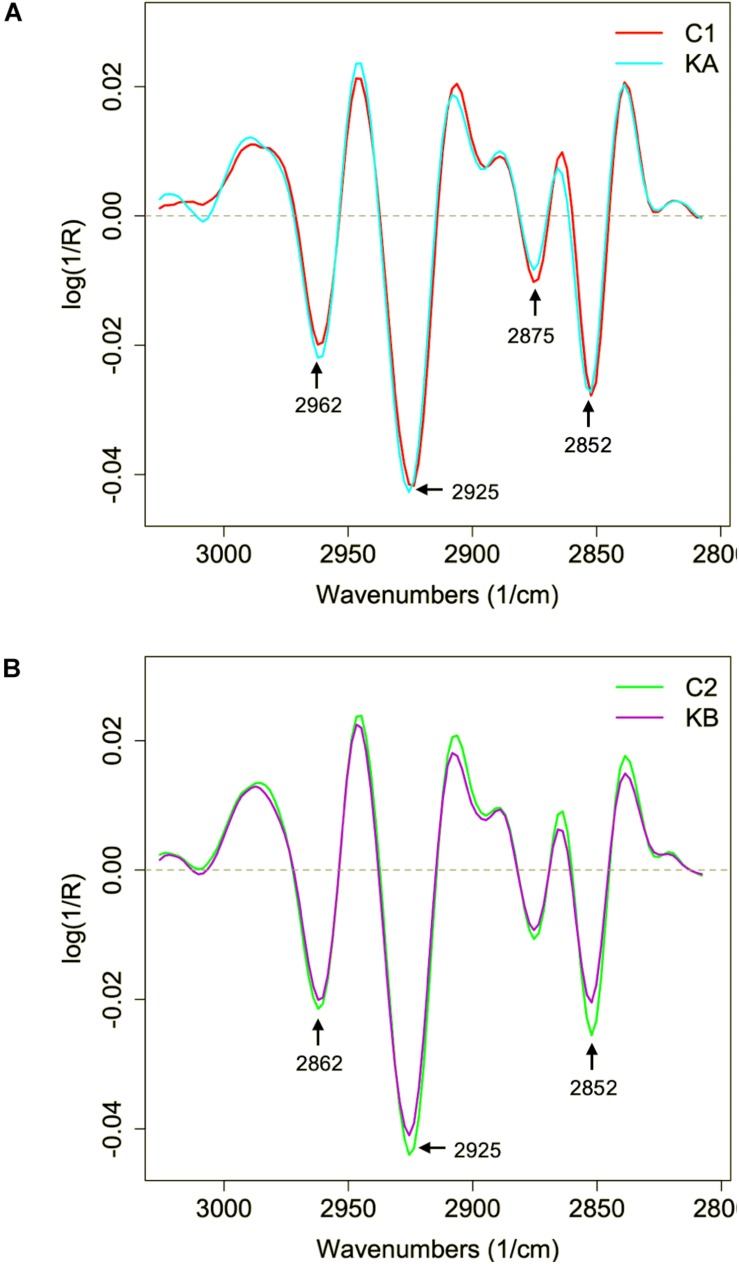
Second derivative of the mean infrared spectral data of the yeast *L. thermotolerans* lipid region before and after dehydration. Cells were dehydrated according to kinetics KA and KB. **(A)** Lipid spectral profile of yeast cells before (red curve, C1) and after dehydration with kinetics KA (cyan curve). **(B)** Lipid spectral profile of yeast cells before (green curve, C2) and after dehydration with kinetics KB (purple curve).

The CH stretching region of the KA and C1 spectra groups were compared by PCA. The score plot for the CH stretching region shows a clustering between C1 and KA samples along PC2 axis (Figure 8A). The main spectral contributions in the loadings in the lipid region of the spectra were the CH_2_ *sym* (∼2840 cm^–1^), CH_2_ *asym* (∼2915 cm^–1^), CH_3_ *sym* (∼2875 cm^–1^), and CH_3_ *asym* (∼2960 cm^–1^) bands (Figure 8A, right). The KA group is elongated on the PC1 axis while the C1 group is compact. PC1 loadings are dominated by peaks at 2933 and 2920 cm^–1^ capturing a shift in the CH_2_ asymmetric stretching peak. This showed that the KA groups exhibited larger dispersion of the CH_2_ peak position associated with more variety in aliphatic lipid conformations. PC1 loadings also captured some changes of the CH_2_ symmetrical stretching at around 2840 cm^–1^ confirming the previous observation. The PC2 loadings are dominated by opposed peaks around 2860 and 2845 cm^–1^, capturing a shift in the CH_2_ symmetric stretching peak position toward lower wavenumbers in the KA group. Indeed, a small 1–2 cm^–1^ upward shift of the CH_2_ symmetric and asymmetric stretching peaks can be observed in Figure 9A.

The C2 and KB spectra were also compared by PCA. The score plot for C2 and KB samples showed a partial separation along PC1 axis (Figure 8B). The PC1 loadings are dominated by opposed peaks at 2916 and 2937 cm^–1^ capturing a widening of the CH_2_ asymmetric stretching peak, and by opposed peaks at 2846 and 2860 cm^–1^ capturing a shift of the CH_2_ symmetric stretching peak.

In both groups, the spectral changes point to changes in the conformation of the lipids. The ordered or disordered state of the lipid membranes are related to the low or high frequencies of the CH_2_ stretching vibrations and correspond, respectively, to their rigid or fluid state (Los and Murata, 2004). Moreover, imaging of CH_2_ symmetric stretching vibrations of the wild-type yeast *S. cerevisiae* BY4742 correlated to the formation of lipid droplets with the CARS (coherent anti-Stokes Raman scattering) technique (Wolinski and Kohlwein, 2015). Thus, modifications observed in the CH_2_ region of yeast *L. thermotolerans* led us to infer that cells changed the nature or the organization of lipids, e.g., short chain fatty acids, more unsaturated fatty acids or formation of lipid droplets, after both dehydration kinetics KA or KB. Indeed, the presence of unsaturated fatty acids has already been correlated with the yeast *S. cerevisiae* higher survival during dehydration (Nguyen et al., 2017) and lactic bacteria during freezing (Passot et al., 2015). This could be related to the higher plasma membrane fluidity (Moussa et al., 2008) which is essential for the maintenance of cell viability after dehydration-rehydration cycles (Dupont et al., 2010).

To further observe the relative lipid/protein content in cells, the spectral bands CH_2_:CH_3_ ratios were calculated. The contribution of the CH_3_ band comes mainly from proteins and the CH_2_ group from lipids. The CH_3_ band was set from 2950 to 2970 cm^–1^ and the CH_2_ band between 2905 and 2945 cm^–1^. After normalization at peak CH_3_ (2960 cm^–1^) this ratio gives information on the lipid content present in the cell and are shown in Table 1. Two groups C1/KA and C2/KB were observed, where the second showed higher CH_2_:CH_3_ ratio than the first, 2.5–2.6 and 2.8, respectively. Therefore, *L. thermotolerans* yeast seems to have expressed a biological variability since the change in relative lipid/protein content did not correlate with dehydration kinetics, but with the samples themselves. However, further studies are needed to confirm these observations, especially under nutrient-limited conditions that lead to lipid droplet formation in *L. thermotolerans* yeast under stress (Câmara et al., 2019b).

**TABLE 1:** CH_2_:CH_3_ ratio of *L. thermotolerans* cells.

	CH_2_:CH_3_ ratio
C1	2.536 ± 0.070^a^
KA	2.628 ± 0.186^a^
C2	2.864 ± 0.270^b^
KB	2.894 ± 0.199^b^

^ab^Different letters on the same column represent significant differences according to Tukey’s HSD (*p* < 0.05). Spectral data normalization was performed at CH_2_ asymmetric peak (2960 cm^–1^).

The CH_3_ bands are related to the stretching vibrations of alkyl group and are associated to the organization of the aliphatic chains of the plasma membrane (Kabelitz et al., 2003). The relationship of CH_3_ band vibration with the resistance of microorganisms to dehydration is less reported in the literature. However, increasing CH_3_ stretching intensity seems to be associated to the relative increase in the amount of lipids or lipid type present in the cell (Jamin et al., 2003). On the one hand, the loss of some molecular components may be related to the increase in the lipids content (Burattini et al., 2008). On the other hand, this higher concentration may be a result of the plasma membrane disorder and fluidity (Grube et al., 2014). These findings suggest that the resistance of *L. thermotolerans* yeast is accompanied by modifications in lipid profiles.

The second derivative of the spectral data in the lipid region of 3050 to 2800 cm^–1^ was studied, which corresponds to the CH groups, before and after dehydration kinetics KA and KB (Figure 9). Significant modifications were observed in the absorption bands in regions 2962, 2925, 2875, and 2852 cm^–1^, which correspond to methyl (CH_3_ *asym*.) and methylene groups (CH_2_ *asym.* and CH_2_ *sym.*). The *L. thermotolerans* cells dehydrated with kinetic KA showed higher intensities of these four main regions compared to the control condition. The CH_2_ and CH_3_ bands, are frequently associated with the membrane fluidity, thus the higher intensity could be an adaptive response of yeast *L. thermotolerans* to dehydration. This observation also explains the ∼44% cell viability after dehydration with kinetics KA, whereas cells dehydrated with kinetics KB showed <10% of cell viability (Figure 4). Indeed, cells dehydrated with kinetics KB showed lower intensity on 2925 cm^–1^ and 2854 cm^–1^ bands compared to the control condition (C2), these regions correspond to vibrations of the CH_2_ and CH_3_ stretching bands (Figure 9B). In this case, modifications in plasma membrane organization may have led to this behavior and was also correlated with the higher cell mortality. It was shown that the decrease in water activity of the cells during dehydration leads to decreasing of plasma membrane fluidity and thus to cell death (Simonin et al., 2008). In addition, cells maintained under kinetics KB conditions may not have had sufficient time to compensate the stress effects of this dehydration condition.

**FIGURE 10:**
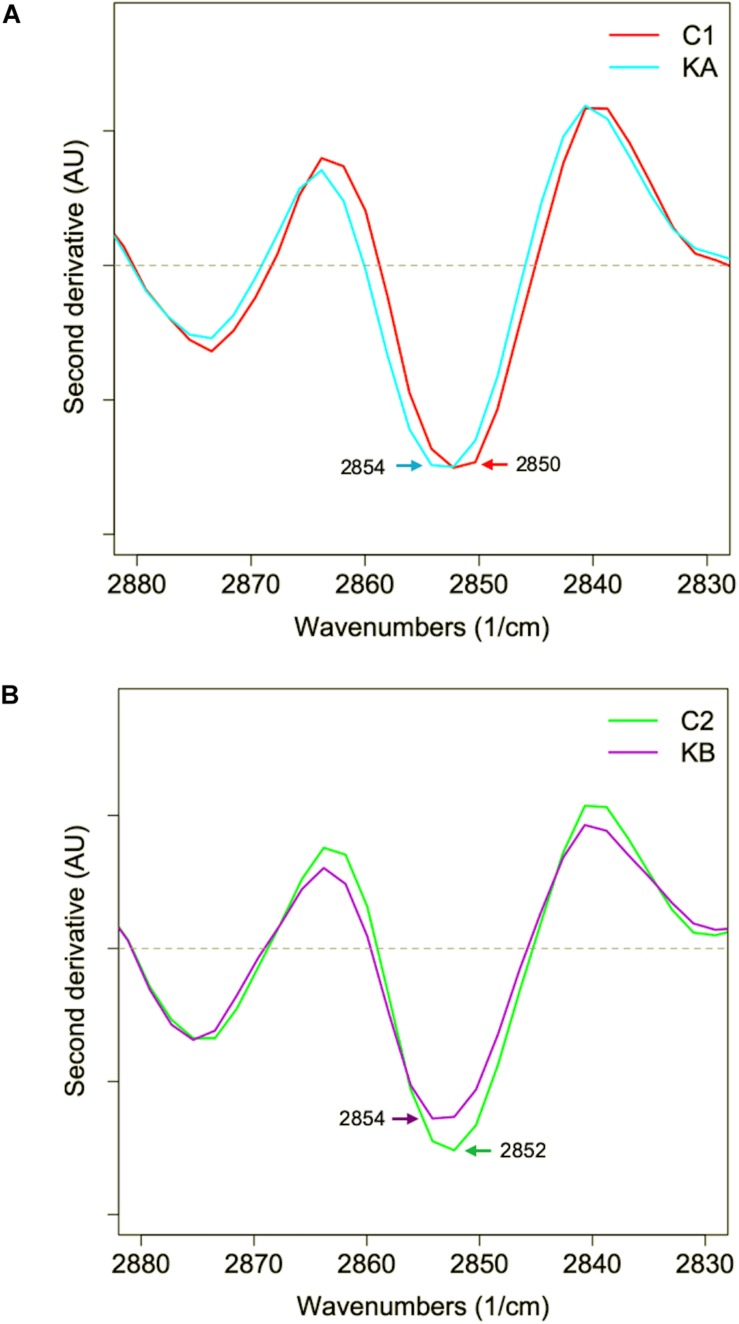
Mean second derivative of the mean infrared spectra of yeast cells in the lipid region (2900–2830 cm^–1^). **(A)** Second derivative for cells before (C1) and after dehydration with kinetics KA. **(B)** Second derivative for cells before (C2) and after dehydration with kinetics KB.

In order to observe changes in the CH_2_ region, which can give an indication of membrane fluidity (de Sarrau et al., 2012), the mean second derivative of cells before and after dehydration with KA and KB kinetics were plotted in Figures 10A,B, respectively. Symmetrical vibration shift of CH_2_ groups was observed when cells were dehydrated with both kinetics and for a higher wavenumber, from 2850 cm^–1^ to 2854 cm^–1^ and from 2852 cm^–1^ to 2854 cm^–1^ for KA and KB, respectively. The shift in the CH_2_ group has already been reported as indicative of lipid aliphatic chain modifications when the bacteria *Lactobacillus delbrueckii* ssp. *bulgaricus* CFL1 was frozen (Passot et al., 2015). However, this same study observed a decrease in wavenumber shift after the frozen treatment, which was correlated with plasma membrane rigidification and loss of cell viability. In this case, the *L. thermotolerans* yeast behavior can be related to increased membrane fluidity after both treatments, but with different magnitudes, where cells dehydrated with KA kinetics showed higher wavenumber shift, therefore better membrane fluidity than KB cells. Indeed, high wavenumbers of symmetric C–H stretching vibration of the CH_2_ indicate decreased lipid acyl chain order (Brandenburg and Seydel, 1998). These findings explain the higher cell viability observed for dehydrated cells with KA kinetics, as well as the loss of cell viability in the KB condition and corroborates the previous observations. This indicates an impact of dehydration conditions on membrane properties of *L. thermotolerans* yeast, where cells dehydrated at a milder temperature has more fluid membranes.

## Conclusion

This was the first time that biophysical responses of *L. thermotolerans* yeast were analyzed during dehydration using synchrotron radiation-based Fourier-transform infrared (S-FTIR) microspectroscopy. It was clearly demonstrated that the maintenance of cellular viability was strongly affected by modifications in the secondary structure of proteins (α-helix and β-sheet) and the shift of stretching vibration of the CH_2_ group, which higher wavenumber suggested a plasma membrane fluidity behavior. On the other hand, the loss of cell viability, especially after dehydration kinetics KB, was affected by the protein degradation or disorder, as well as by the increased plasma membrane rigidity. For the first time, it was demonstrated an in-depth biophysical study of the yeast *L. thermotolerans* during dehydration. These findings are of substantial importance for understanding the mechanisms of microbial resistance to dehydration and serve as a basis for optimizing this sensitive yeast industrial production.

## Data Availability Statement

The datasets generated for this study are available on request to the corresponding author.

## Author Contributions

AC, RS, and FH designed the study. AC, TN, RS, CS, and FH performed the experiments. AC, CP, LD, CS, and FH performed the data analysis. AC and FH wrote the manuscript. CP, LD, and CS reviewed the manuscript. All authors read and approved the final manuscript.

## Conflict of Interest

CS was employed by the company Synchrotron SOLEIL. The remaining authors declare that the research was conducted in the absence of any commercial or financial relationships that could be construed as a potential conflict of interest.
